# Better health-related quality of life in kidney transplant patients compared to chronic kidney disease patients with similar renal function

**DOI:** 10.1371/journal.pone.0257981

**Published:** 2021-10-04

**Authors:** Jung-Hwa Ryu, Tai Yeon Koo, Han Ro, Jang-Hee Cho, Myung-Gyu Kim, Kyu Ha Huh, Jae Berm Park, Sik Lee, Seungyeup Han, Jayoun Kim, Kook-Hwan Oh, Jaeseok Yang, KNOW-KT Study group

**Affiliations:** 1 Transplantation Center, Seoul National University Hospital, Seoul, Republic of Korea; 2 Nephrology, Ewha Womans University Seoul Hospital, Seoul, Republic of Korea; 3 Nephrology, Seongnam Citizens Medical Center, Seongnam, Republic of Korea; 4 Department of Internal Medicine, Gachon University Gil Medical Center, Incheon, Republic of Korea; 5 Department of Internal Medicine, Kyungpook National University Hospital, Daegu, Republic of Korea; 6 Department of Internal Medicine, Korea University College of Medicine, Seoul, Republic of Korea; 7 Department of Surgery, Yonsei University College of Medicine, Seoul, Republic of Korea; 8 Department of Surgery, Seoul Samsung Medical Center, Sungkyunkwan University, Seoul, Republic of Korea; 9 Department of Internal Medicine, Chonbuk National University Hospital, Jeonju, Republic of Korea; 10 Department of Internal Medicine, Keimyung University, Dongsan Medical Center, Daegu, Republic of Korea; 11 Medical Research Collaborating Center, Seoul National University Hospital, Seoul, Republic of Korea; 12 Department of Internal Medicine, Seoul National University College of Medicine, Seoul, Republic of Korea; 13 Division of Nephrology, Department of Internal Medicine, Yonsei University College of Medicine, Seoul, Republic of Korea; Istituto Di Ricerche Farmacologiche Mario Negri, ITALY

## Abstract

Renal functional deterioration is associated with physical and mental burdens for kidney transplant (KT) and chronic kidney disease (CKD) patients. However, the change in health-related quality of life (HRQOL) over time in KT patients compared to that of native CKD patients has not been evaluated. We addressed this issue using KT patients registered in the KNOW-KT cohort study and patients at CKD stage 1–3 registered in the KNOW-CKD cohort study. HRQOL scores were assessed using the Kidney Disease Quality of Life Short Form at baseline, 2-, and 4-years follow-up in 842 KT patients and at baseline and 5-year follow-up in 1,355 CKD patients. SF-36 scores declined at the 4-year follow-up, whereas CKD-targeted scores showed no change in the KT group. In contrast, CKD-targeted scores as well as SF-36 scores were decreased at the 5-year follow-up in CKD patients. When prognostic factors were analyzed for longitudinal HRQOL data over time, renal functions, diabetes, cardiovascular and cerebrovascular diseases, hemoglobin level, marital status, income, employment, and health care were significant prognostic factors. Furthermore, KT was an independent prognostic factor for better HRQOL. These results highlight that KT can offer a better HRQOL than that of CKD patients, even when renal function is similar.

## Introduction

Chronic kidney disease (CKD) patients suffer a worsening quality of life (QOL) along with an increased risk of morbidity and mortality as their renal function deteriorates progressively [1, 2]. CKD patients, including dialysis patients, experience significant physical and mental burdens such as fatigue, pruritus, sleep disturbance, pain, depression, and restless leg syndrome [3–6], ultimately leading to an impaired physical and psycho-social health status [7]. This poor QOL has been strongly associated with adverse clinical outcomes such as increased mortality, morbidity, and frequent hospitalization in CKD patients [1, 8, 9]. In this sense, assessment of health-related QOL (HRQOL) is useful to estimate the health status, disease burden, treatment effectiveness, and even survival in CKD patients [10, 11]. Previous studies have shown that in CKD patients, HRQOL is positively associated with the estimated glomerular filtration rate (eGFR) level [12, 13], and deteriorates in line with progression through CKD stages [14]. Moreover, native CKD patients show higher QOL scores than hemodialysis patients but still have lower QOL scores than the general population [15].

The two main goals of kidney transplantation (KT) are improvements in survival and QOL, and the latter has received increased research attention in recent years. Several studies have shown that patients who underwent successful KT had better HRQOL as well as improved survival compared to patients who received hemodialysis and peritoneal dialysis [16–18]. Furthermore, a lower HRQOL in KT patients was independently associated with post-transplant mortality and morbidity. These findings suggest the importance of regular HRQOL assessments in KT patients [19].

KT patients are considered to be a subgroup of the CKD population, since they have only a single functional kidney and the recovered renal function also slowly declines over time [20]. The post-KT eGFR is frequently below 60 mL·min^-1^·1.73 m^-^2 and one report suggested that 32.3% of KT patients belonged to CKD stage 3b (eGFR <45 mL·min^-1^·1.73 m^-^2) 1 year after transplantation [21]. Despite studies on changes in HRQOL over time in CKD patients, no longitudinal study of QOL changes beyond 1 year after KT has been performed for this important CKD subgroup. Moreover, the HRQOL patterns in KT patients and native CKD patients with similar renal function have not been compared.

Therefore, the aim of this study was to investigate the longitudinal changes in HRQOL with respect to both general health status and CKD-specific health status in KT patients, and to compare these HRQOL patterns in KT and CKD patients at the same CKD stages. For this analysis, we used data of patients enrolled in the KoreaN cohort study for Outcome in patients With Kidney Transplantation (KNOW-KT) and the KoreaN cohort study for Outcome in patients With Chronic Kidney Disease (KNOW-CKD) [22, 23].

## Materials and methods

### Ethics statement

This study protocol was approved by the Institutional Review Board at Seoul National University College of Medicine/Seoul National University Hospital (H-1901-124-1005) and was conducted in accordance with the 1964 Declaration of Helsinki and its later amendments or comparable ethical standards. All participants provided informed written consent.

### Study design and participants

KNOW-KT and KNOW-CKD are ongoing multicenter, observational, cohort studies of Korean KT and native CKD patients. The detailed study design of each cohort has previously been published [22, 23]. In brief, the KNOW-KT study enrolled and followed up adult living- or deceased-donor KT patients annually, while the KNOW-CKD study enrolled and followed up adult native CKD patients according to Kidney Disease Improving Global Outcomes Clinical Practice Guideline annually [20]. Because of the relatively small number of KT patients at CKD stage 4–5, data of patients at CKD stages 4 and CKD 5 were excluded. A total of 1,080 KT patients from eight Korean transplantation centers and 2,238 CKD patients from nine clinical centers were registered in these studies from 2011 to 2016. Those who had never participated in the HRQOL questionnaire test were excluded from the analysis. The eGFR was calculated by four-variable Modification of Diet in Renal Disease equations [24]. Primary outcome is QOL at baseline and follow-up time points. Main exposures are kidney transplantation vs. CKD, renal functions (eGFR), and follow-up time.

### Data collection

Clinical information, including socio-demographic, medical histories, anthropometric, and laboratory data, at baseline and at follow-up was reviewed. Demographic variables included age, gender, marital status, monthly income, employment, and level of education. Higher education was defined as graduation from college or graduate school. Higher income was defined as a monthly family income higher than US $4,500. Employment was determined by the individual work status at the time of questionnaire request. Higher education level was defined as a final educational level of college diploma or above. Medical demographic data included primary cause of CKD, diabetes, hypertension, cardiovascular disease (CVD), and cerebrovascular disease. CVD was defined as heart failure or coronary arterial disease requiring hospitalization and coronary intervention. Anthropometric measurements were performed for height, weight, and body mass index (BMI), calculated as weight/height2 (kg/m2). The laboratory parameters were hemoglobin, serum albumin, and serum creatinine. As KT group-specific factors, the type of native dialysis modality, dialysis duration, donor type, and immunosuppressive regimens were included in the analysis.

### Assessment of HRQOL

QOL was assessed by self-administered questionnaires using the Kidney Disease Quality of Life Short Form (KDQOL-SF, version 1.3) [25]. This questionnaire consists of a kidney-specific part, CKD-targeted scores, and a general part, termed SF-36 [26, 27]. The KDQOL-SF has been validated for estimation of QOL in the Korean population and the KT population [26–28] as a multidimensional tool, which makes it possible to conduct subdomain comparisons. In brief, the CKD-targeted scores contain 43 kidney-specific items that are categorized into 11 subdomains: symptoms/problems, effects of kidney disease, burden of kidney disease, work status, cognitive function, quality of social interaction, sexual function, sleep, social support, encouragement by the dialysis staff, and patient satisfaction with care. The SF-36 scores consist of 36 items that belong to eight subdomains divided into the physical component summary (PCS) scores and mental component summary (MCS) scores. The survey generates scores on 8 scales, 4 scales in PCS and MCS, respectively. PCS includes physical functioning, role limitations due to physical health, bodily pain, general health, and MCS includes vitality (energy), social functioning, role limitations due to emotional problems, and mental health (emotional wellbeing). Responses to each item were transformed into KDQOL-SF equivalent scores ranging from 0 to 100-point linear scores, with higher scores indicating better QOL. Self-administration is the principle of this assessment; however, the questionnaire was completed with the help of a study assistant when a patient had difficulty doing so. Assessment of HRQOL using KDQOL-SF was performed at 2 years after KT as the baseline and at the 2-year and 4-year follow-up in the KT group. HRQOL assessment was performed at baseline and at the 5-year follow-up in the CKD group.

### Statistical analysis

Continuous variables were expressed as the mean ± standard deviation or median (interquartile range), and categorical variables were expressed as number (percentage). The Student t-test, Mann Whitney U test, or chi-square test was used for comparison, as appropriate. We analyzed prognostic factors associated with higher HRQOL scores that were repeatedly-measured over multiple time-points. For this longitudinal statistical analysis over time, we used a generalized estimated equation (GEE).

Follow-up time from baseline was included as time variable in this model. Multivariate model included all covariates yielding unadjusted P value less than 0.2 as well as clinical important variables such as age, gender, and eGFR without a selection algorithm. Two-sided *P* values less than 0.05 were considered statistically significant. All statistical analyses were performed using SPSS for Windows version 25.0 (IBM Corp., Armonk, NY, USA).

## Results

### Changes in HRQOL after KT

**Fig 1:**
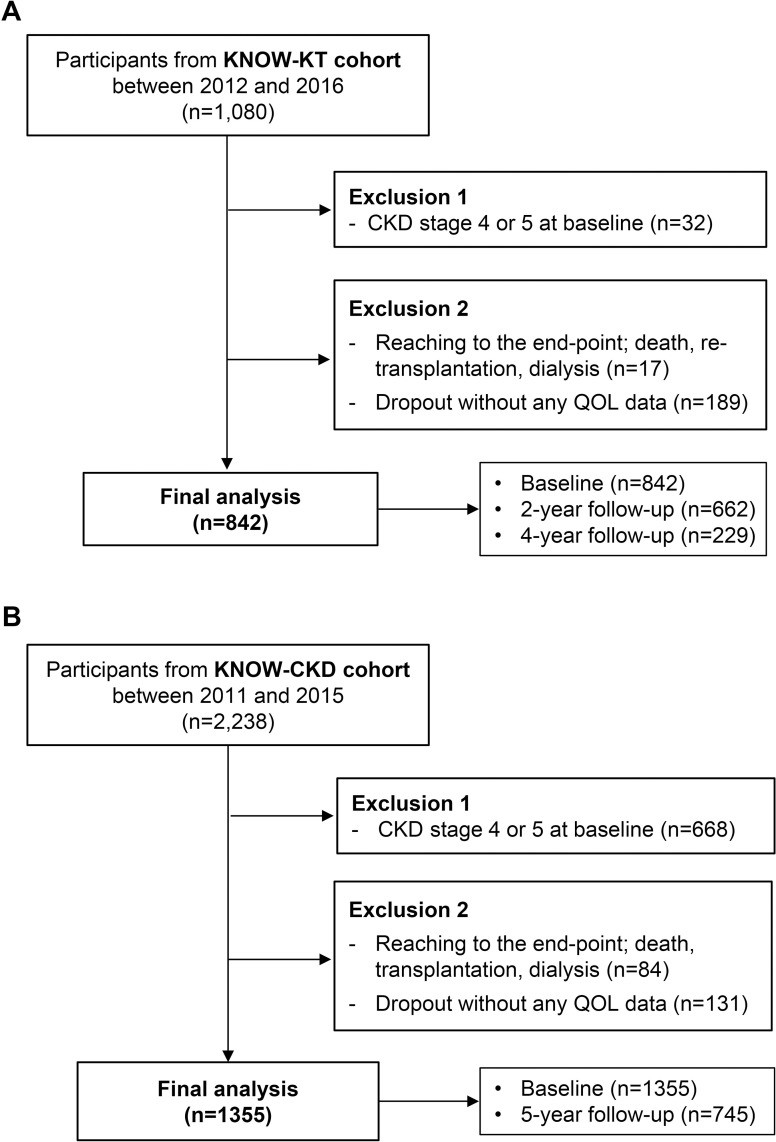
Flow diagram of the study population. (A) Among 1,080 KNOW-KT cohort participants, total 842 patients were analyzed after exclusion of 238 people. (B) Among 2,238 KNOW-CKD cohort participants, total 1,355 patients were analyzed after exclusion of 883 people. KNOW-KT, KoreaN cohort study for Outcome in patients With Kidney Transplantation; QOL, quality of life; KNOW-CKD, KoreaN cohort study for Outcome in patients With Chronic Kidney Disease.

A total of 842 KT patients in the KNOW-KT cohort who had available baseline HRQOL data at 2 years after KT or follow-up HRQOL data, were included in the final analysis (Fig 1A).

When baseline characteristics were compared between included subjects and excluded subjects among KT patients with CKD stage 1–3 at baseline, there were no significant differences in the clinical characteristics except for age between the two groups (S1 Table). The clinical and laboratory characteristics of the final analysis set are summarized in Table 1. At the 2-year follow-up, eGFR did not differ from baseline, whereas at the 4-year follow-up eGFRs were significantly lower than baseline eGFRs. Both hemoglobin levels and albumin levels were significantly lower at the 4-year follow-up compared to baseline.

**Table 1:** Comparison of clinical characteristics between KT and CKD patients at baseline.

Variables	CKD (N = 1355)	KT (N = 842)	*P* *
Age (years, mean ± SD)	52.5 ± 12.5	45.3 ± 11.7	0.001
Male gender (%)	829 (61.2%)	531 (63.1%)	0.391
Marriage (%)	1106 (81.6%)	615 (73.0%)	0.034
Education (%)			
College or post-graduate	638 (47.1%)	429 (51.0%)	0.250
Economy (%)			
High (> $ 4,500/ month)	333 (24.6%)	131 (15.6%)	0.134
Current employment (%)	826 (61.6%)	426 (50.6%)	0.021
Health insurance (%)	1238 (91.4%)	781 (92.8%)	0.184
BMI (kg/m^2^, mean ± SD)	24.6 ± 3.4	22.6 ± 3.2	0.001
Cause of ESRD (%)			0.014
DM	218 (16.1%)	161 (19.1%)	
HTN	223 (16.5%)	246 (29.2%)	
GN	545 (40.2%)	269 (31.9%)	
ADPKD	290 (21.4%)	44 (5.2%)	
Others	79 (5.8%)	122 (14.5%)	
DM	334 (24.6%)	201 (23.8%)	0.487
Hypertension	999 (73.7)	775 (92.0%)	0.020
Cardiovascular disease	85 (6.3%)	48 (5.7%)	0.395
Cerebrovascular disease	69 (5.1%)	29 (3.4%)	0.972
eGFR (mL/min/1.73 m^2^, mean ± SD)			
Baseline	63.3 ± 27.2	66.0 ± 17.0	0.009
Hemoglobin (g/dL, mean ± SD)			
Baseline	13.5 ± 1.9	13.9 ± 1.9	0.001
Albumin (g/dL, mean ± SD)			
Baseline	4.2 ± 0.4	4.4 ± 0.3	0.001

ADPKD, autosomal dominant polycystic kidney disease; BMI, body mass index; DM, diabetes mellitus; eGFR, estimated glomerular filtration rate by MDRD equation; ESRD, end-stage renal disease; GN, glomerulonephritis; HD, hemodialysis; HTN, hypertension; SD, standard deviation.

*P < 0.05, CKD patients vs. KT patients (Paired t-test).

**Fig 2:**
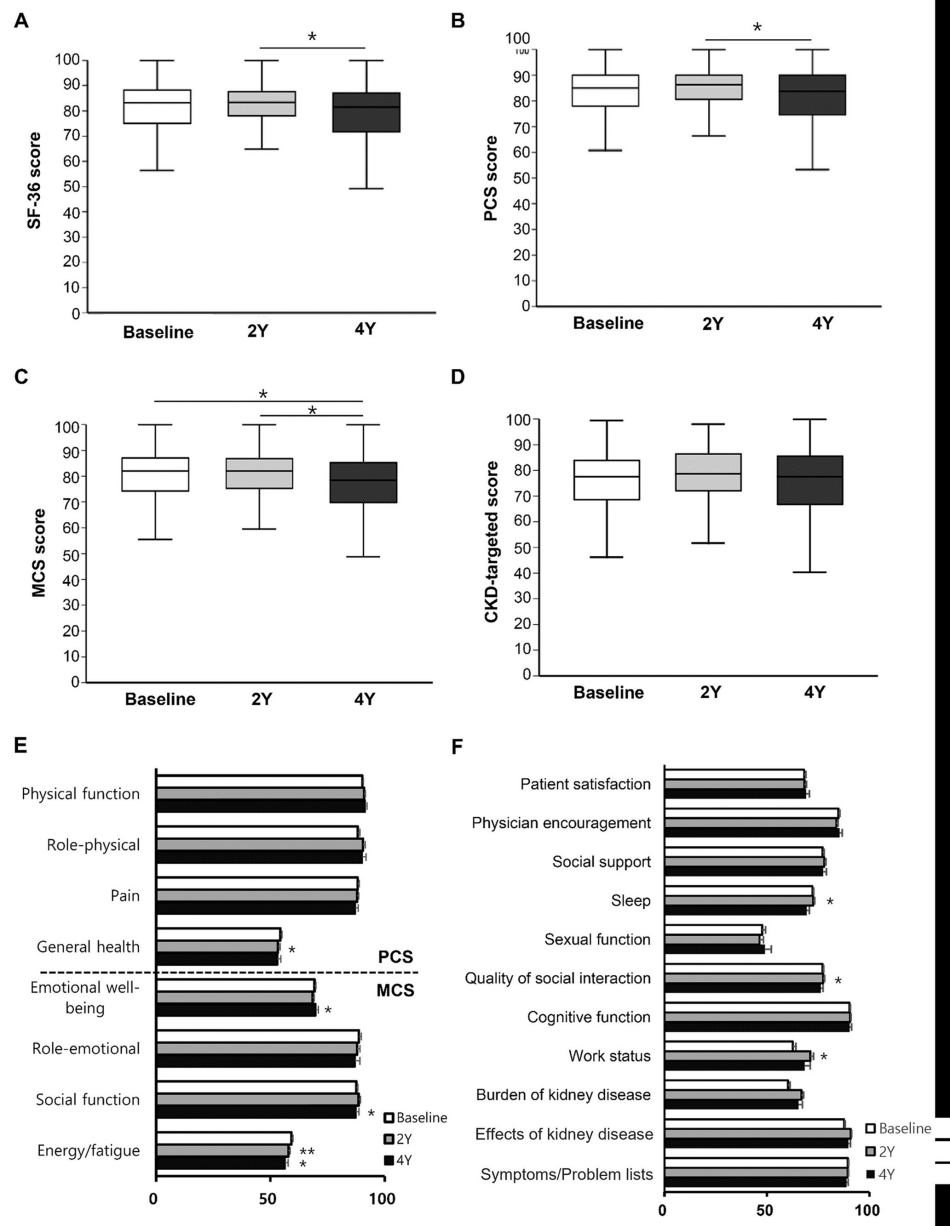
Changes in SF-36 scores and CKD-targeted scores after kidney transplantation. (A) HRQOL at baseline, 2- and 4-year follow-up was assessed by SF-36 scores in kidney transplant patients. (B-C) Physical component summary (PCS) score (B) and mental component summary (MCS) score (C) were also assessed. (D) Total CKD-targeted score was assessed by KDQOL-SF at baseline, 2- and 4-year follow-up. All values in panel a-d were displayed using Box and whisker plots. Top, middle, and bottom of boxes were the 75th, 50th, and 25th percentiles, respectively; whiskers illustrate the range. (E, F) Each domain covering PCS or MCS scores in SF-36 scores (E) and the CKD-targeted scores (F) was separately analyzed. Each value in panel e and f was displayed as the mean ± standard error of the mean. *P<0.05 and **P<0.01 compared to baseline (paired t-test). CKD, chronic kidney disease; HRQOL, health-related quality of life; KDQOL-SF, Kidney Disease Quality of Life Short Form; SF-36, Short Form-36 Health Survey (SF-36).

The mean HRQOL scores were calculated at baseline, at the 2-year follow-up, and at the 4-year follow-up. The SF-36 scores, including both physical and mental QOLs and CKD-targeted scores did not show significant changes at the 2-year follow-up (Fig 2). However, SF-36 scores had decreased significantly at the 4-year follow-up (Fig 2A). Both physical and mental QOLs had decreased significantly at the 4-year follow-up (-2.5 points and -2.3 points, respectively) (Fig 2B and 2C). In contrast, the CKD-targeted score did not change during follow-up (Fig 2D). In the subdomain analysis of PCS, general health showed lower values at the 2-year follow-up than at baseline. Among MCS categories, emotional well-being, social function, and energy/fatigue showed significantly decreased values at the 4-year follow-up (Fig 2E). With respect to the subdomain analysis for CKD-target scores, compositional scores showed inconsistent changes. The scores for work status, quality of social interaction, and sleep were slightly decreased at the 4-year follow-up (Fig 2F).

When we classified patients to group of decline, no change, or improvement according to QOL changes, decline, no change, and improvement of SF-36 score were found in 110 (56.1%), 3 (1.5%), and 83 (42.3%) patients, respectively. For CKD-targeted score, decline, no change, improvement was found in 110 (56.1%), 3 (1.5%), and 83 (42.3%) patients, respectively.

### Prognostic factors associated with better HRQOL in KT recipients

We analyzed independent prognostic factors associated with higher HRQOL scores at the 4-year follow-up. Analysis using a GEE revealed that absence of diabetes mellitus, higher blood hemoglobin level, higher income, married status, and current employment were associated with higher SF-36 scores (Table 2). Younger age, absence of hypertension, absence of diabetes mellitus, higher eGFR, higher income, married status, and current employment were associated with higher CKD-targeted scores (Table 2).

**Table 2:** Prognostic factors associated with HRQOL in KT patients ^a^.

Parameter	Higher SF-36 score			Higher CKD-targeted score		
	Estimate (95% C.I.)	SE	*P*	Estimate (95% C.I.)	SE	*P*
Time	-0.603 (-3.281, 1.276)	1.109	0.169	-0.193 (-2.243, 1.813)	1.042	0.848
Age	-0.081 (-0.189, 0.078)	0.057	0.154	-0.124 (-2.124, 3.439)	0.048	0.009
Gender (Male)	0.270 (-1.412, 1.402)	1.182	0.819	2.381 (0.318, 4.444)	1.053	0.024
Hypertension	-2.358 (-7.432, 2.273)	2.399	0.326	-3.575 (-6.931, -0.222)	1.712	0.037
Diabetes mellitus	-5.207 (-7.889, -2.396)	1.275	< 0.001	-2.659 (-4.651, -0.534)	1.063	0.012
Cardiovascular Ds	-3.229 (-7.538, 1.654)	2.124	0.128	-1.177 (-4.538, 2.183)	1.770	0.492
Cerebrovascular Ds	-0.579 (-6.388, 5.231)	2.872	0.845	-1.152 (-5.546, 2.393)	2.394	0.630
BMI	-0.068 (-0.349, 0.226)	0.155	0.659	-0.092 (-0.407, 0.189)	0.129	0.476
eGFR	0.030 (-0.037, 0.076)	0.025	0.372	0.053 (0.008, 0.097)	0.021	0.020
Albumin	1.500 (-2.316, 5.317)	1.215	0.247	0.657 (-2.124, 3.439)	1.012	0.648
Hemoglobin	0.627 (0.108, 1.291)	0.346	0.020	0.241 (-0.249, 0.731)	0.289	0.335
Marriage	3.471 (0.208, 5.315)	1.364	0.011	4.304 (1.832, 6.038)	1.137	<0.001
Higher education ^b^	0.982 (-1.368, 3.002)	1.109	0.376	-1.222 (-2.958, 0.594)	0.924	0.186
Higher income ^c^	3.367 (1.493, 6.015)	1.267	0.010	2.527 (1.210, 4.285)	1.056	0.017
Employment	2.629 (0.356, 4.687)	1.105	0.017	5.911 (3.989, 7.616)	0.921	<0.001
Health insurance (vs. Health care)	1.678 (-3.202, 5.912)	2.017	0.560	1.051 (-2.477, 4.580)	1.681	0.559

BMI, body mass index; C.I., confidence interval; CKD, chronic kidney disease; Ds, Disease; eGFR, estimated glomerular filtration rate by MDRD equation; KT, kidney transplantation; SE, standard error.

^a^ Generalized estimated equation analysis was performed.

^b^ Higher education was defined as receiving a diploma from college or higher.

^c^ Higher income was defined as monthly income above $ 4,500. *P* value by generalized estimated equation analysis.

### Changes in HRQOL over time in CKD patients

A total of 1,355 CKD patients in the KNOW-CKD cohort with available HRQOL data at baseline or follow-up HRQOL data were included in this study (Fig 1B). When baseline characteristics were compared between included subjects and excluded subjects among patients with CKD stage 1–3 at baseline, the excluded patients were older than the included patients. Diabetes and hypertension were more frequent in the excluded group (S2 Table). The clinical and laboratory characteristics of the final analysis set are described in Table 1.

In the CKD population, renal function decreased significantly over time (64.3 ± 26.6 mL·min-1·1.73 m-2 at baseline vs. 57.3 ± 28.4 mL·min-1·1.73 m-2 at 5 years; *P* = 0.001). In addition, both the SF-36 and CKD-targeted scores were decreased at the 5-year follow-up (S1A and S1D Fig). The mean changes in QOL scores between baseline and the 5-year follow-up were higher for CKD-targeted scores than for SF-36 (-1.5 and -0.85 points, respectively). Physical QOL was also significantly decreased at 5 years compared to the baseline levels (S1B Fig). However, mental QOL showed no significant difference between baseline and the 5-year follow-up (S1C Fig). Among the SF-36 subdomains, scores for role limitations due to physical problems (-3.2 points) were most remarkably decreased (S1E Fig). With respect to the CKD-target score, sexual dysfunction (-8.2 points), impaired work status (-4.6 points), kidney-disease effects (-4.3 points), and sleep (-2.8 points) were markedly decreased at the 5-year follow-up (S1F Fig).

Decline, no change, and improvement of SF-36 score during follow up were found in 392 (53.9%), 10 (1.4%), and 325 (44.7%) patients, respectively. Decline, no change, and improvement of the CKD-targeted score were found in 423 (58.2%), 0%, and 304 (41.8%) patients, respectively.

### Prognostic factors associated with better HRQOL in CKD patients

Analysis using a GEE revealed that absence of diabetes, absence of cerebrovascular disease, higher hemoglobin level, male gender, married status, higher education, higher income, current employment, and health insurance were independently associated with higher SF-36 scores at the 5-year follow-up in the CKD patients (S3 Table). Absence of diabetes, absence of cerebrovascular disease, higher eGFR at baseline, higher blood hemoglobin level, male gender, married status, higher income, current employment, and health insurance were independently associated with higher CKD-targeted scores at the 5-year follow-up (S3 Table).

### Comparison of HRQOL changes in KT and CKD populations

**Fig 3:**
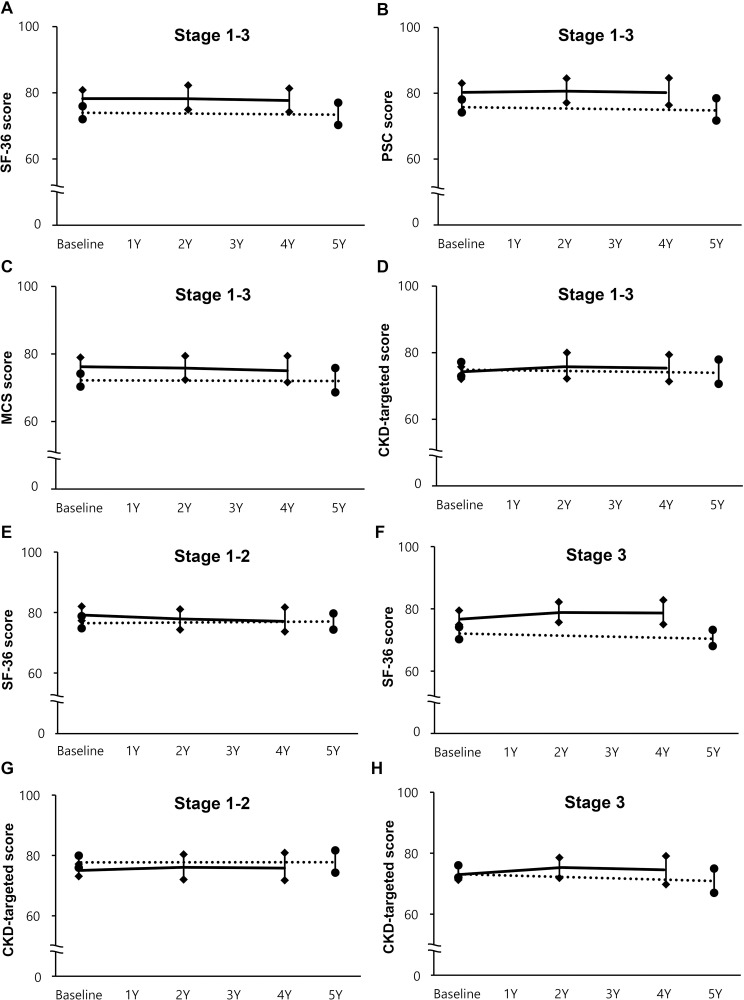
Comparison of changes in HRQOL between KT and CKD patients at the similar renal function according to CKD stage. (A) SF-36 score at CKD stage 1–3. (B) PCS score at CKD stage 1–3. (C) MCS score at CKD stage 1–3. (D) CKD-targeted score at CKD stage 1–3. (E) SF-36 score at CKD stage 1–2. (F) SF-36 score at CKD stage 3. (G) CKD-targeted score at CKD stage 1–2. (H) CKD-targeted score at CKD stage 3 were compared between KT (solid lines) and CKD patients (dot lines). Each value was displayed as the mean ± standard error of the mean. Chronic kidney disease; MCS, mental component summary score; KT, kidney transplantation; PCS, Physical component summary score.

Next, we compared the HRQOL changes over time in KT and native CKD patients. The baseline, 2-, and 4-year follow-up data of KT patients were compared with the baseline and 5-year data of CKD patients (Fig 3A–3D). When clinical characteristics were compared between the KT and CKD group (Table 1), the CKD patients were significantly older than the KT patients (52.5 ± 12.5 vs. 45.3 ± 11.7 years, *P* = 0.001). The SF-36 scores were higher in KT patients than in CKD patients at baseline (78.2 ± 12.8 vs. 73.9 ± 15.6, *P* = 0.001), whereas the CKD-targeted scores were similar between the two groups at baseline (74.3 ± 12.0 vs. 74.9 ± 12.3, *P* = 0.261). During follow-up, SF-36 scores as well as PCS scores and MCS scores were higher in the KT population than in the CKD population during the study period (Fig 3A–3C). However, the CKD-targeted score over time remained similar between the two groups, although the difference seemed to increase over time (Fig 3D).

A GEE analysis of the combined population of KT and CKD patients also demonstrated that KT was significantly associated with a higher SF-36 score (*P* < 0.0001) along with absence of diabetes, absence of CVD, absence of cerebrovascular disease, higher hemoglobin level, married status, higher education, higher family income, current employment, and health insurance (Table 3 and Fig 3A). Renal function (eGFR) showed a borderline significance for SF-36 (*P* = 0.058, Table 3). However, KT was not associated with higher CKD-targeted scores (*P* = 0.561). Instead, renal function (*P* = 0.003) was a significant prognostic factor for CKD-targeted scores along with male gender, absence of diabetes, absence of CVD, absence of cerebrovascular disease, higher hemoglobin level, married status, higher family income, current employment, and health insurance (Table 3).

**Table 3:** Prognostic factors associated with HRQOL in the total population including both KT and CKD patients ^a^.

Parameter	Higher SF-36 score			Higher CKD-targeted score		
	Estimate (95% C.I)	SE	*P*	Estimate (95% C.I.)	SE	*P*
KT (vs. CKD)	8.760 (5.290, 9.414)	1.041	< .0001	0.428 (-1.102, 1.941)	0.735	0.561
Time	-1.457 (-3.585, 1.408)	1.450	0.315	-0.462 (-2.902, 2.289)	1.333	0.729
eGFR	0.028 (-0.055, 0.058)	0.015	0.058	0.037 (0.011, 0.058)	0.012	0.003
Age	-0.011 (-0.085, 0.028)	0.034	0.753	-0.031 (-0.064, 0.026)	0.027	0.236
Gender (Male)	0.032 (-1.358, 1.422)	0.789	0.083	1.024 (0.085, 2.134)	0.566	0.040
Hypertension	-1.367 (-3.035, 0.714)	1.054	0.195	-0.156 (-1.495, 1.082)	0.818	0.849
Diabetes mellitus	-4.662 (-5.829, -2.501)	0.881	< .0001	-3.035 (-4.127, -1.353)	0.683	< .0001
Cardiovascular Ds	-3.683 (-6.471, -0.257)	1.561	0.018	-2.634 (-4.537, -0.195)	1.211	0.030
Cerebrovascular Ds	-4.121 (-7.405, -1.120)	1.787	0.021	-3.511 (-6.578, -0.903)	1.387	0.011
BMI	-0.079 (-0.292, 0.134)	0.109	0.524	-0.047 (-0.222, 0.127)	0.089	0.594
Albumin	1.357 (-0.754, 3.467)	1.076	0.208	1.381 (-0.147, 3.015)	0.705	0.075
Hemoglobin	1.153 (0.544, 2.337)	0.230	< .0001	0.704 (0.423, 1.047)	0.178	< .0001
Marriage	3.585 (1.766, 5.341)	1.021	0.0004	4.818 (3.411, 6.190)	0.792	< .0001
Higher education ^b^	2.965 (1.000, 3.750)	0.771	0.010	0.085 (-0.981, 1.150)	0.598	0.876
Higher income ^c^	3.249 (1.980, 4.725)	0.904	0.0003	2.164 (1.414, 3.735)	0.701	0.002
Employment	3.899 (2.243, 5.203)	0.789	< .0001	6.731 (5.384, 7.613)	0.612	< .0001
Health insurance (vs. Health care)	4.962 (1.313, 9.083)	1.657	0.003	3.602 (0.569, 5.899)	1.285	0.017

BMI, body mass index; C.I., confidence interval; CKD, chronic kidney disease; Ds, Disease; eGFR, estimated glomerular filtration rate by MDRD equation; KT, kidney transplantation; SE, standard error.

^a^ Generalized estimated equation analysis was performed.

^b^Higher education was defined as receiving a diploma from college or higher.

^c^ Higher income was defined as monthly income above $ 4,500. *P* value by generalized estimated equation analysis.

### Comparison of HRQOL changes in KT and CKD populations according to CKD stages

In patients with CKD stage 1–2, higher baseline SF-36 scores in the KT patients decreased slightly, there were no changes in the scores of CKD patients, and the scores of the two groups were more similar at the later part of follow-up (Fig 3E and S4 Table). In contrast, the difference in SF-36 scores between stage 3 KT patients and CKD patients increased during follow-up (GEE, interaction of group and time, *P* = 0.031, Fig 3F and S5 Table).

CKD-targeted scores also showed different change patterns over time according to baseline CKD stages. There was no significant difference in CKD-targeted scores over time between KT and CKD patients irrespective of CKD stage (Fig 3G and 3H). However, in CKD stage 3, the difference in the CKD-targeted scores seemed to increase over time (GEE, interaction of group and time, *P* = 0.009, Fig 3H and S5 Table).

## Discussion

The present cohort study demonstrated that KT patients had higher SF-36 scores with respect to both physical and mental QOL than CKD patients at the same CKD stage; however, they had similar CKD-targeted scores compared to CKD patients with similar renal function. KT was a significant prognostic factor associated with better QOL, independent of renal function.

CKD patients suffer from a low QOL [1, 2]. The AUSDiab analysis reported that physical QOL in CKD patients decreased in parallel with eGFR decline during a 5-year follow-up [29]. Consistently, our study showed that SF-36 scores, mainly PCS scores decreased over 5 years. The CKD-targeted score also decreased along with decreasing renal function, and better renal function was independently associated with higher CKD-targeted QOL, in line with previous studies [7, 12, 29]. A Korean community-based analysis also showed that QOL was inversely correlated with CKD stage [30]. Taken together, these findings clearly demonstrate that QOL in CKD patients becomes worse along with decreasing renal function, which is an important prognostic factor associated with QOL in CKD patients.

KT immediately improves the renal function of ESRD patients. A Greek study demonstrated improvement in QOL components such as better general health perception, role of emotional function, and vitality among the subdomains of the SF-36 score in KT patients 1 year after transplantation [31]. A recent US study reported deterioration of physical QOL and a stationary CKD-targeted score at 1 month after KT, but significant improvements in physical and CKD-targeted QOL at 3 months, especially for more frail patients [32]. We also previously reported significant improvements in HRQOL for both SF-36 and CKD-targeted QOL scores at 2 years after KT [33]. Furthermore, the present follow-up study of a larger group demonstrated that the improved QOL at 2 years after KT began to decline at 4-year follow-up in the KT group.

Previous studies have assessed the association between QOL at the early phase with long-term clinical outcomes such as mortality and allograft function in KT populations. Higher physical and mental health scores are significant prognostic factors for better 10-year survival [34], and lower physical function and general health perception in the physical component are independent risk factors for 7-year mortality [19]. We hope that further long-term follow-up studies using our KT cohort can demonstrate the impact of QOL at each time-point and its influence on the long-term clinical outcomes after KT.

We investigated the prognostic factors associated with QOL in KT and CKD cohorts. A single-center study in Thailand reported that gender, marital status, higher income, and higher education were associated with increased QOL after KT [35]. In the present study, absence of diabetes mellitus, higher blood hemoglobin level, higher monthly income, marriage, and employment were independently associated with higher SF-36 scores in KT or CKD patients. The CKD-targeted score was significantly associated with decreased renal function, absence of diabetes, higher income, employment, and married status in both the KT and CKD groups. The hemoglobin level consistently influenced QOL in KT and CKD patients [36–38]. Diabetes was another important prognostic factor influencing QOL in the present KT population in parallel with reports of non-KT populations [39]. These findings suggest that efforts are needed to improve QOL during the pre-KT CKD phase to achieve a better post-KT QOL along with correction of anemia.

Because KT patients remain in a state of CKD, we tried to explore differences in QOL between KT and non-KT CKD patients with similar renal function. Previous studies demonstrated that KT patients had better QOL than ESRD patients undergoing dialysis [17, 40–43]. The overall improvement in QOL after KT was mostly attributed to improvement of physical function [44]. However, few studies have compared QOL between KT patients and native CKD patients. A small-sized, single-center study that compared 38 stable KT recipients and 38 CKD patients at CKD stage 3b–4 showed no significant difference in the SF-36 QOL score, but a better QOL in non-dialysis CKD patients than in KT patients based on a visual analogue scale [45]. In contrast, in the present multicenter study, KT patients had higher SF-36 QOL scores than the CKD patients after adjustment for other factors, including renal function. These positive effects of KT on QOL were observed in various domains of the MCS and in the general health domain of the PCS. When we analyzed the impact of KT on QOL over time compared to CKD according to CKD stages, SF-36 scores were higher in KT patients than in CKD patients at CKD stage 3 as well as CKD stages 1–2. On the other hand, CKD-targeted scores in KT and CKD patients did not differ at baseline and there was no significant difference in the change of CKD-targeted scores over time between the two groups irrespective of CKD stage. Interestingly, the difference in both SF-36 and CKD-targeted scores between the two groups increased over time at CKD stage 3, suggesting that the beneficial effects of KT on HRQOL compared to CKD might increase with CKD progression. Overall, KT patients had better SF-36 scores than CKD patients independent of renal function, whereas the CKD-targeted score was mainly dependent on renal function and KT did not provide additional benefits in patients with similar renal function.

Some of baseline characteristics except renal functions, such as age, BMI, albumin levels, hemoglobin levels, marriage status, and current employment, were different between the KT group and the CKD group. However, the KT group showed better QOL than the CKD group after adjustment of these different factors by multivariate analysis.

The reasons why KT patients have better QOL than CKD patients with similar renal function are unclear. KT patients might interpret their QOL more positively than CKD patients, which may be related to their history of improvement with KT and lower levels of concern about future prognosis compared to CKD patients without a dramatic recovery experience. Indeed, depression was found to be the most important factor influencing HRQOL in the CKD population [46]. Depressive mood consists of lack of enthusiasm, feelings of hopelessness for the future, feeling left behind in society, and an associated feeling of worthlessness, which may result in a poor QOL at advanced CKD stages. In contrast, the KT population considered the possibility of recovering renal function with treatment or retransplantation, which might bring hope and reduce depressive mood. The higher QOL in KT patients might allay fears related to disease progression so as to conform to the CKD status.

This study had several limitations. First, the number of patients at the 4-year follow-up was relatively small in the KT population. Second, this study did not include advanced CKD stage 4–5 patients because of low numbers, so we could not compare KT and CKD patients at this advanced stage. Therefore, further longer-term follow-up studies using this cohort could help to more adequately assess the association between renal functional deterioration and QOL changes in KT patients, and the impact of KT on QOL compared to CKD in advanced CKD stages. Third, unfortunately the follow-up time-points of serial QOL assessment were different in the KT and CKD cohorts. Furthermore, KT patients have had longer exposure time to chronic kidney disease than native CKD patients and cannot exclude a possibility that different exposure time might have influenced different QOL between CKD with transplanted kidneys and CKD with native kidneys at similar renal functions. Future comparative studies with the same follow-up schedule are needed to confirm our findings. Fourth, we did not include healthy subjects with normal kidney function in this study. Further study to compare QOL between the healthy control and the KT group could estimate benefits in QOL in the KT group more clearly. Finally, we used MDRD equation to estimate eGFR in both native CKD and KT population. However, MDRD equation is not a gold standard to calculate eGFR in KT patients. Some data showed CKD-EPI equation has least bias to evaluate eGFR in KT population compared to MDRD [47]. However, because CKD-EPI method overestimates eGFR in CKD stage 1 and 2, MDRD method was recommended as a good tool to assess eGFR in KT population [48]. Furthermore, MDRD equation was accurate and least bias in other studies [49]. Because no calculation method for eGFR in transplant patients perfectly represents measured GFR in whole range of GFR, application of the same tools in two groups could be reasonable.

Despite these limitations, to the best of our knowledge, this prospective, longitudinal HRQOL study represents the longest follow-up QOL assessment of KT patients at multiple time-points. Furthermore, this is the first study to compare QOL between KT and CKD patients with similar renal function and a mild to moderate degree of CKD (stage 1–3). Based on this study, further studies using other cohorts could confirm our findings.

In conclusion, KT patients have better QOL than CKD patients with similar renal function at CKD stages 1–3. Therefore, improvement in QOL is an important benefit of KT for CKD patients.

## Supporting information

S1 Fig Changes in SF-36 scores and CKD-targeted scores in CKD patients. (A) HRQOL at baseline and 5-year follow-up was assessed by SF-36 scores in CKD patients. (B-C), Physical component summary (PCS) score (B) and mental component summary (MCS) score (C) were also assessed. (D)Total CKD-targeted score was assessed by KDQOL-SF at baseline and 5-year follow-up. All values in panel a-d were displayed using Box and whisker plots. Top, middle, and bottom of boxes were the 75th, 50th, and 25th percentiles, respectively; whiskers illustrate the range. (E-F) Each domain covering PCS or MCS scores in SF-36 scores (E) and the CKD-targeted scores (F) was separately analyzed. Each value in panel e and f was displayed as the mean ± standard error of the mean. *P<0.05 compared to baseline values (paired t-test). CKD, chronic kidney disease; KDQOL-SF, Kidney Disease Quality of Life Short Form; SF-36, Short Form-36 Health Survey (SF-36).
(TIF)

S1 Table Clinical characteristics of the enrolled and the excluded KT patients. (DOCX)

S2 Table Clinical characteristics of the enrolled and the excluded CKD patients. (DOCX)

S3 Table Prognostic factors associated with HRQOL in CKD patients. (DOCX)

S4 Table Prognostic factors associated with HRQOL in the total population including both KT and CKD patients at CKD stage 1–2. (DOCX)

S5 Table Prognostic factors associated with HRQOL in the total population including both KT and CKD patients at CKD stage 3. (DOCX)
