# Supplementary figures and images for: Better health-related quality of life in kidney transplant patients compared to chronic kidney disease patients with similar renal function
Source: PLoS One. 2021 Oct 4;16(10):e0257981. doi: 10.1371/journal.pone.0257981 (PMC8489710; doi:10.1371/journal.pone.0257981)

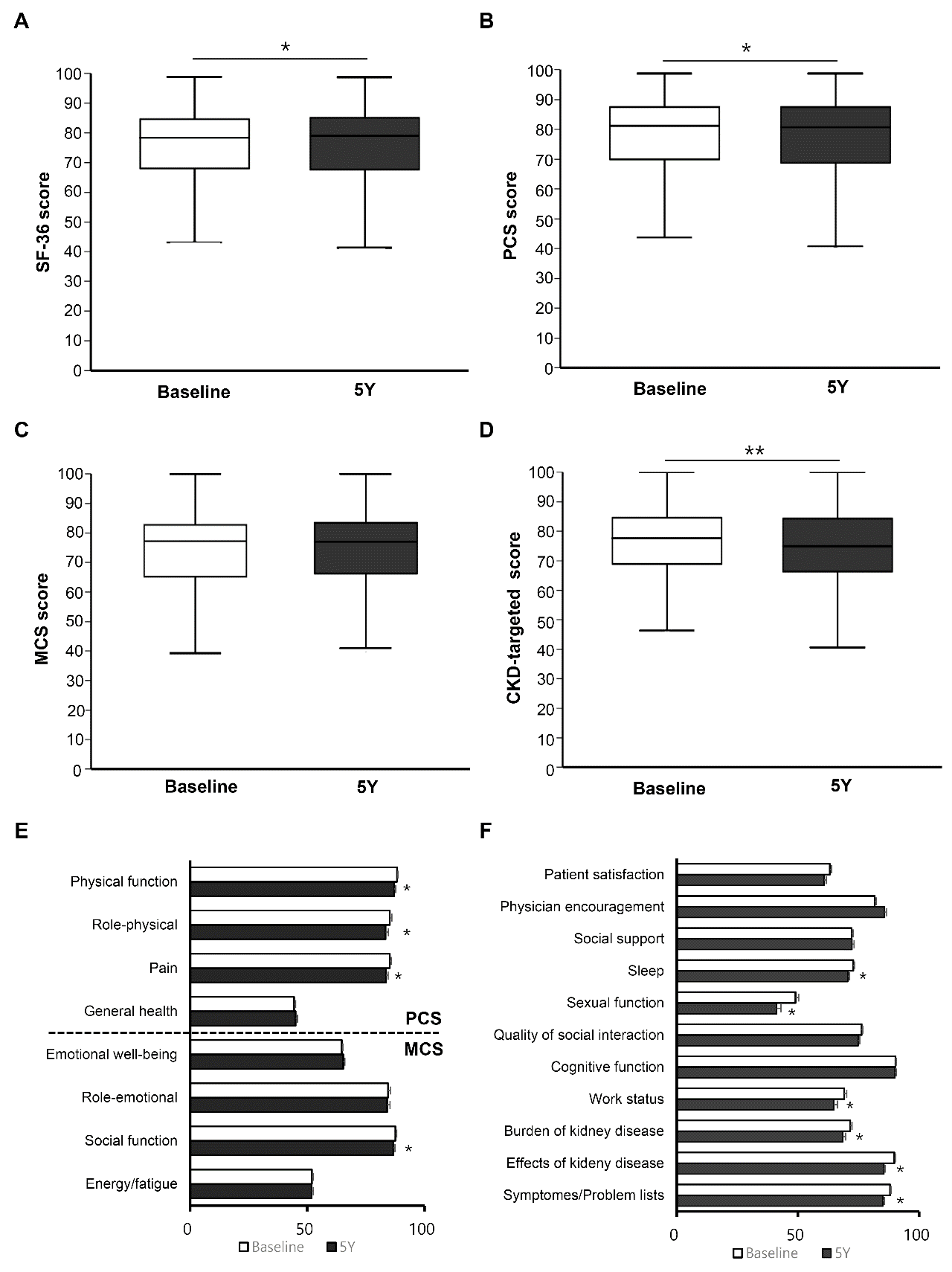

Supplement: S1 Fig — (A) HRQOL at baseline and 5-year follow-up was assessed by SF-36 scores in CKD patients. (B-C), Physical component summary (PCS) score (B) and mental component summary (MCS) score (C) were also assessed. (D)Total CKD-targeted score was assessed by KDQOL-SF at baseline and 5-year follow-up. All values in panel a-d were displayed using Box and whisker plots. Top, middle, and bottom of boxes were the 75th, 50th, and 25th percentiles, respectively; whiskers illustrate the range. (E-F) Each domain covering PCS or MCS scores in SF-36 scores (E) and the CKD-targeted scores (F) was separately analyzed. Each value in panel e and f was displayed as the mean ± standard error of the mean. *P<0.05 compared to baseline values (paired t-test). CKD, chronic kidney disease; KDQOL-SF, Kidney Disease Quality of Life Short Form; SF-36, Short Form-36 Health Survey (SF-36). (TIF) [file pone.0257981.s001.tif]
