# Supplementary material for: Better health-related quality of life in kidney transplant patients compared to chronic kidney disease patients with similar renal function
Source: PLoS One. 2021 Oct 4;16(10):e0257981. doi: 10.1371/journal.pone.0257981 (PMC8489710; doi:10.1371/journal.pone.0257981)
Supplement: S1 Table — (DOCX) [file pone.0257981.s002.docx]

**S1 Table. Clinical characteristics of the enrolled and the excluded KT patients ^a^**

| **Variables** | **Enrolled (N = 842)** | **Excluded (N=206)** | ***P*** |
| --- | --- | --- | --- |
| Age (years, mean ± SD) | 45.3 ± 11.7 | 47.8 ± 11.8 | 0.023^#^ |
| Male gender (%) | 531 (63.1%) | 139 (67.4%) | 0.702 |
| Marriage (%) | 615 (73.0%) | 159 (77.4%) | 0.527 |
| Education (%) |  |  |  |
| College or post-graduate | 429 (51.0%) | 100 (48.5%) | 0.617 |
| Economy (%) |  |  |  |
| High (≥ $ 4,500/month) | 131 (15.6%) | 28 (13.6%) | 0.335 |
| Current employment (%) | 426 (50.6%) | 103 (50.0%) | 0.852 |
| Health insurance (%) | 781 (92.8%) | 183 (88.8%) | 0.213 |
| BMI (kg/m^2^, mean ± SD) | 22.9 ± 3.2 | 22.8 ± 3.5 | 0.914 |
| Cause of ESRD (%) |  |  | 0.227 |
| DM | 161 (19.1%) | 57 (27.7%) |  |
| HTN | 246 (29.2%) | 44 (21.4%) |  |
| GN | 269 (31.9%) | 62 (30.1%) |  |
| ADPKD | 44 (5.2%) | 18 (8.7%) |  |
| Others | 122 (14.5%) | 25 (12.1%) |  |
| Type of RRT (%) |  |  |  |
| HD | 542 (64.5%) | 142 (68.9%) | 0.578 |
| PD | 104 (12.4%) | 21 (10.2%) |  |
| Transplantation | 13 (1.5%) | 4 (1.9%) |  |
| Preemptive | 182 (21.6%) | 39 (18.9%) |  |
| Duration of previous dialysis (month ^b^) | 9.0 (1.0 - 63.5) | 7.9 (1.0 – 51.2) | 0.414 |
| DM | 201 (23.8%) | 67 (32.5%) | 0.062 |
| Hypertension | 775 (92.0%) | 187 (90.8%) | 0.794 |
| Cardiovascular disease | 48 (5.7%) | 22 (10.7%) | 0.105 |
| Cerebrovascular disease | 29 (3.4%) | 5 (2.4%) | 0.284 |
| Type of donor (%) |  |  |  |
| Living | 694 (82.4%) | 167 (81.8%) | 0.847 |
| Desensitization (%) | 216 (25.7%) | 57 (27.7%) | 0.625 |
| eGFR (mL/min/1.73 m^2^, mean ± SD) |  |  |  |
| Baseline | 66.0 ± 17.0 | 65.2 ± 20.5 | 0.628 |
| 2-year follow-up | 65.6 ± 19.2 | 64.2 ± 25.8 | 0.715 |
| 4-year follow-up | 62.2 ± 19.8* | 68.4 ± 34.8* | 0.221 |
| Hemoglobin (g/dL) |  |  |  |
| Baseline | 13.9 ± 1.9 | 13.5 ± 2.4 | 0.318 |
| 2-year follow-up | 13.8 ± 1.9 | 13.3 ± 2.3 | 0.335 |
| 4-year follow-up | 13.6 ± 1.8* | 13.8 ± 2.5* | 0.498 |
| Albumin (g/dL) |  |  |  |
| Baseline | 4.4 ± 0.3 | 4.4 ± 0.3 | 0.502 |
| 2-year follow-up | 4.3 ± 0.3 | 4.5 ± 0.3 | 0.437 |
| 4-year follow-up | 4.3 ± 0.3* | 4.5 ± 0.3 | 0.421 |
| Immunosuppressant (%) |  |  |  |
| Tacrolimus among CNI | 796 (94.5%) | 198 (96.1%) | 0.902 |
| Mycophenolate mofetil~~e~~ | 815 (96.8%) | 199 (96.6%) | 0.947 |
| mTOR inhibitors | 78 (9.3%) | 22 (8.8%) | 0.895 |
| Steroid | 831 (98.7%) | 204 (99.0%) | 0.938 |

ADPKD, autosomal dominant polycystic kidney disease; BMI, body mass index; CNI, calcineurin inhibitor; DM, diabetes mellitus; eGFR, estimated glomerular filtration rate by MDRD equation; ESRD, end-stage renal disease; GN, glomerulonephritis; HD, hemodialysis; HTN, hypertension; KT, kidney transplantation; mTOR, mammalian target of rapamycin; PD, peritoneal dialysis; RRT, renal replacement therapy; SD, standard deviation. **P* < 0.05 compared to baseline. ^#^*P* < 0.05 at comparison between the enrolled and the excluded groups. ^a^ KT patients with CKD stage 1–3 at baseline. ^b^ Median (range).
