# Supplementary material for: Better health-related quality of life in kidney transplant patients compared to chronic kidney disease patients with similar renal function
Source: PLoS One. 2021 Oct 4;16(10):e0257981. doi: 10.1371/journal.pone.0257981 (PMC8489710; doi:10.1371/journal.pone.0257981)
Supplement: S2 Table — (DOCX) [file pone.0257981.s003.docx]

**S2 Table. Clinical characteristics of the enrolled and the excluded CKD patients ^a^**

| **Variables** | **Enrolled (N = 1355)** | **Excluded (N=215)** | ***P*** |
| --- | --- | --- | --- |
| Age (years, mean ± SD) | 52.5 ± 12.5 | 56.7 ± 12.5 | 0.001^#^ |
| Male gender (%) | 829 (61.2%) | 128 (59.5%) | 0.692 |
| Marriage (%) | 1106 (81.6%) | 163 (79.1%) | 0.601 |
| Education (%) |  |  |  |
| College or post-graduate | 638 (47.1%) | 95 (44.2%) | 0.427 |
| Economy (%) |  |  |  |
| High (> $ 4,500/ month) | 333 (24.6%) | 50 (23.3%) | 0.689 |
| Current employment (%) | 826 (61.6%) | 131 (60.9%) | 0.527 |
| Health insurance (%) | 1238 (91.4%) | 194 (90.2%) | 0.495 |
| BMI (kg/m^2^, mean ± SD) | 24.6 ± 3.4 | 25.1 ± 3.7 | 0.325 |
| Cause of ESRD (%) |  |  | 0.086 |
| DM | 218 (16.1%) | 51 (23.7%) |  |
| HTN | 223 (16.5%) | 48 (22.3%) |  |
| GN | 545 (40.2%) | 85 (39.5%) |  |
| ADPKD | 290 (21.4%) | 22 (10.2%) |  |
| Others | 79 (5.8%) | 9 (4.1%) |  |
| DM | 334 (24.6%) | 96 (44.7%) | 0.021^#^ |
| Hypertension | 999 (73.7) | 183 (85.1%) | 0.032^#^ |
| Cardiovascular disease | 85 (6.3%) | 30 (14.0%) | 0.089 |
| Cerebrovascular disease | 69 (5.1%) | 20 (9.3%) | 0.235 |
| eGFR (mL/min/1.73 m^2^, mean ± SD) |  |  |  |
| Baseline | 64.3 ± 26.6 | 61.1 ± 28.1 | 0.112 |
| 5-year follow-up | 57.3 ± 28.4* | 53.1 ± 26.1 | 0.423 |
| Hemoglobin (g/dL, mean ± SD) |  |  |  |
| Baseline | 13.5 ± 1.9 | 13.3 ± 2.1 | 0.228 |
| 5-year follow-up | 13.2 ± 1.9 | 13.0 ± 2.3 | 0.231 |
| Albumin (g/dL, mean ± SD) |  |  |  |
| Baseline | 4.2 ± 0.4 | 4.2 ± 0.4 | 0.718 |
| 5-year follow-up | 4.2 ± 0.4 | 4.1 ± 0.4 | 0.108 |

ADPKD, autosomal dominant polycystic kidney disease; BMI, body mass index; eGFR, estimated glomerular filtration rate by MDRD equation; DM, diabetes mellitus; GN, glomerulonephritis; HTN, hypertension; SD, standard deviation. **P* < 0.05 compared to baseline. ^#^*P* < 0.05 at comparison between the enrolled and the excluded groups. ^a^ CKD Patients with CKD stage 1–3 at baseline.
