# Supplementary material for: Better health-related quality of life in kidney transplant patients compared to chronic kidney disease patients with similar renal function
Source: PLoS One. 2021 Oct 4;16(10):e0257981. doi: 10.1371/journal.pone.0257981 (PMC8489710; doi:10.1371/journal.pone.0257981)
Supplement: S3 Table — (DOCX) [file pone.0257981.s004.docx]

**S3 Table. Prognostic factors associated with HRQOL in CKD patients ^a^**

| **Parameter** | **Higher SF-36 score** | | |  | **Higher CKD-targeted score** | | |
| --- | --- | --- | --- | --- | --- | --- | --- |
|  | **Estimate (95% C.I.)** | **SE** | ***P*** |  | **Estimate (95% C.I.)** | **SE** | ***P*** |
| Time | -0.883 (-1.990, 0.499) | 0.634 | 0.240 |  | -0.644 (-2.059, 0.219) | 0.469 | 0.225 |
| Age | 0.033 (-0.021, 0.112) | 0.025 | 0.449 |  | 0.026 (-0.024, 0.076) | 0.025 | 0.604 |
| Gender (Male) | 2.599 (0.228, 3.125) | 0.655 | 0.017 |  | 1.627 (0.471, 2.291) | 0.642 | 0.039 |
| Hypertension | -2.113 (-4.449, -0.659) | 0.967 | 0.008 |  | -1.092 (-2.718, 0.515) | 0.886 | 0.406 |
| Diabetes mellitus | -3.712 (-6.091, -1.808) | 1.093 | 0.003 |  | -2.428 (-3.810, -0.676) | 0.893 | 0.007 |
| Cardiovascular Ds | -2.851 (-5.660, 0.737) | 2.117 | 0.209 |  | -3.264 (-6.416, -0.835) | 1.424 | 0.011 |
| Cerebrovascular Ds | -5.225 (-9.455, -0.702) | 2.488 | 0.025 |  | -5.160 (-8.372, -1.257) | 1.815 | 0.008 |
| BMI | 0.023 (-0.234, 0.280) | 0.131 | 0.861 |  | 0.021 (-0.144, 0.133) | 0.763 | 0.642 |
| eGFR | 0.002 (-0.031, 0.036) | 0.017 | 0.893 |  | 0.040 (0.017, 2.753) | 0.014 | 0.005 |
| Albumin | 1.230 (-1.290, 3.629) | 1.254 | 0.868 |  | 1.897 (1.135, 3.343) | 0.989 | 0.035 |
| Hemoglobin | 1.213 (0.675, 1.730) | 0.323 | <.0001 |  | 1.089 (0.742, 32.435) | 0.234 | <.0001 |
| Marriage | 4.571 (2.772, 7.061) | 1.251 | 0.003 |  | 5.999 (4.048, 7.501) | 0.881 | <.0001 |
| Higher education ^b^ | 3.856 (2.154, 5.559) | 0.869 | <.0001 |  | 0.883 (-0.369, 2.135) | 0.639 | 0.167 |
| Higher income ^c^ | 2.787 (0.859, 4.290) | 0.875 | 0.029 |  | 1.853 (0.546, 3.352) | 0.716 | 0.006 |
| Employment | 5.620 (3.332, 7.118) | 0.966 | <.0001 |  | 7.281 (5.730, 8.452) | 0.695 | <.0001 |
| Health insurance (vs. Health care) | 10.220 (6.348, 17.293) | 2.734 | <.0001 |  | 6.978 (4.171, 10.398) | 1.982 | <.0001 |

BMI, body mass index; C.I., confidence interval; CKD, chronic kidney disease; Ds, Disease; eGFR, estimated glomerular filtration rate by MDRD equation; KT, kidney transplantation; SE, standard error. ^a^ Generalized estimated equation analysis was performed. ^b^ Higher education was defined as receiving a diploma from college or higher. ^c^ Higher income was defined as monthly income above $ 4,500. *P* value by generalized estimated equation analysis.
