# Supplementary material for: Better health-related quality of life in kidney transplant patients compared to chronic kidney disease patients with similar renal function
Source: PLoS One. 2021 Oct 4;16(10):e0257981. doi: 10.1371/journal.pone.0257981 (PMC8489710; doi:10.1371/journal.pone.0257981)
Supplement: S4 Table — (DOCX) [file pone.0257981.s005.docx]

**S4 Table. Prognostic factors associated with HRQOL in the total population including both KT and CKD patients at CKD stage 1‒2 ^a^**

| **Parameter** | **Higher SF-36 score** | | |  | **Higher CKD-targeted score** | | |
| --- | --- | --- | --- | --- | --- | --- | --- |
|  | **Estimate (95% C.I)** | **SE** | ***P*** |  | **Estimate (95% C.I.)** | **SE** | ***P*** |
| KT (vs. CKD) | 5.032 (2.354, 6.720) | 0.999 | <.0001 |  | 0.043 (-1.619, 1.705) | 0.848 | 0.959 |
| Time | -0.457 (-4.012, 1.418) | 1.450 | 0.315 |  | -0.480 (-3.054, 2.093) | 1.313 | 0.714 |
| eGFR | 0.015 (-0.151, 0.055) | 0.025 | 0.903 |  | 0.042 (0.008, 0.076) | 0.017 | 0.015 |
| Age | -0.038 (-0.115, 0.040) | 0.042 | 0.341 |  | -0.018 (-0.077, 0.041) | 0.030 | 0.546 |
| Gender (Male) | 1.460 (-0.165, 3.575) | 0.658 | 0.120 |  | 1.387 (-0.090, 2.864) | 0.754 | 0.066 |
| Hypertension | -1.539 (-3.618, 0.673) | 0.894 | 0.174 |  | 0.661 (-1.022, 2.344) | 0.859 | 0.442 |
| Diabetes mellitus | -3.525 (-5.518, -0.802) | 0.763 | 0.009 |  | -1.044 (-2.871, 0.783) | 0.932 | 0.263 |
| Cardiovascular Ds | -4.771 (-9.751, 0.944) | 2.728 | 0.107 |  | -2.999 (-6.645, 0.648) | 0.932 | 0.107 |
| Cerebrovascular Ds | -1.611 (-7.013, 3.883) | 2.779 | 0.557 |  | -3.617 (-7.676, 0.442) | 2.072 | 0.081 |
| BMI | -0.045 (-0.297, 0.208) | 0.111 | 0.487 |  | -0.060 (-0.283, 0.163) | 0.114 | 0.599 |
| Albumin | 2.255 (-0.843, 5.363) | 0.944 | 0.147 |  | 1.630 (-0.514, 3.775) | 1.094 | 0.136 |
| Hemoglobin | 0.705 (0.251, 1.390) | 0.187 | 0.015 |  | 0.679 (0.242, 1.116) | 0.223 | 0.002 |
| Marriage | 4.197 (1.779, 6.501) | 1.187 | 0.001 |  | 5.245 (3.394, 7.097) | 0.945 | <.0001 |
| Higher education ^b^ | 0.457 (-1.594, 2.146) | 0.948 | 0.631 |  | 0.810 (-0.621, 2.325) | 0.750 | 0.280 |
| Higher income ^c^ | 4.629 (2.515, 6.221) | 0.929 | <.0001 |  | 3.061 (1.459, 4.662) | 0.815 | 0.001 |
| Employment | 1.499 (-0.890, 2.945) | 0.978 | 0.294 |  | 5.621 (4.071, 7.171) | 0.788 | <.0001 |
| Health insurance (vs. Health care) | 5.874 (-0.661, 10.928) | 2.892 | 0.083 |  | 3.525 (-0.467, 7.684) | 2.085 | 0.091 |

BMI, body mass index; C.I., confidence interval; CKD, chronic kidney disease; Ds, Disease; eGFR, estimated glomerular filtration rate by MDRD equation; KT, kidney transplantation; SE, standard error. ^a^ Generalized estimated equation analysis was performed. ^b^ Higher education was defined as receiving a diploma from college or higher. ^c^ Higher income was defined as monthly income above $ 4,500. *P* value by generalized estimated equation analysis.
