# Supplementary material for: Better health-related quality of life in kidney transplant patients compared to chronic kidney disease patients with similar renal function
Source: PLoS One. 2021 Oct 4;16(10):e0257981. doi: 10.1371/journal.pone.0257981 (PMC8489710; doi:10.1371/journal.pone.0257981)
Supplement: S5 Table — (DOCX) [file pone.0257981.s006.docx]

| **Parameter** | **Higher SF-36 score** | | |  | **Higher CKD-targeted score** | | |
| --- | --- | --- | --- | --- | --- | --- | --- |
|  | **Estimate (95% C.I.)** | **SE** | ***P*** |  | **Estimate (95% C.I.)** | **SE** | ***P*** |
| KT (vs. CKD) | 5.032 (2.935, 7.128) | 1.067 | <.0001 |  | 0.412 (-1.490, 2.315) | 0.971 | 0.808 |
| Time | -0.579 (-2.201, 1.043) | 0.827 | 0.570 |  | -1.525 (-5.112, 2.062) | 1.081 | 0.406 |
| eGFR | -0.028 (-0.137, 0.081) | 0.247 | 0.612 |  | 0.016 (-0.066, 0.099) | 0.042 | 0.690 |
| Age | 0.025 (-0.062, 0.112) | 0.042 | 0.569 |  | -0.026 (-0.092, 0.041) | 0.034 | 0.451 |
| Gender (Male) | -1.376 (-3.79, 0.661) | 1.039 | 0.186 |  | 0.708 (-0.922, 2.338) | 0.832 | 0.395 |
| Hypertension | -0.336 (-3.464, 2.793) | 1.596 | 0.883 |  | 0.191 (-2.141, 2.522) | 0.191 | 0.873 |
| Diabetes mellitus | -4.609 (-6.938, -2.280) | 1.188 | <.0001 |  | -4.080 (-5.801, -2.360) | 0.878 | <.0001 |
| Cardiovascular Ds | -2.852 (-6.488, 0.783) | 1.855 | 0.124 |  | -2.079 (-4.699, 0.541) | 1.337 | 0.120 |
| Cerebrovascular Ds | -5.555 (-10.798, -0.312) | 2.675 | 0.038 |  | -3.934 (-7.922, 0.054) | 2.035 | 0.053 |
| BMI | -0.265 (-0.598, 0.068) | 0.170 | 0.119 |  | -0.036 (-0.303, 0.230) | 0.136 | 0.789 |
| Albumin | 0.842 (-1.997, 3.682) | 1.449 | 0.561 |  | 1.605 (-0.346, 1.231) | 1.605 | 0.107 |
| Hemoglobin | 1.128 (0.564, 1.692) | 0.288 | <.0001 |  | 0.788 (0.346, 1.231) | 0.226 | <.0001 |
| Marriage | 2.809 (0.006, 5.612) | 1.430 | 0.05 |  | 4.219 (2.069, 6.368) | 1.097 | <.0001 |
| Higher education ^b^ | 4.445 (2.477, 6.412) | 1.004 | <.0001 |  | 1.127 (-0.427, 2.680) | 0.793 | 0.155 |
| Higher income ^c^ | 1.862 (-0.157, 3.880) | 1.029 | 0.071 |  | 1.930 (0.220, 3.640) | 0.815 | 0.027 |
| Employment | 5.844 (3.803, 7.884) | 0.665 | <.0001 |  | 7.491 (5.913, 9.069) | 0.805 | <.0001 |
| Health insurance (vs. Health care) | 4.399 (-0.924, 9.723) | 2.716 | 0.150 |  | 2.969 (-0.638, 6.374) | 1.789 | 0.109 |

**S5 Table. Prognostic factors associated with HRQOL in the total population including both KT and CKD patients at CKD stage 3 ^a^**

BMI, body mass index; C.I., confidence interval; CKD, chronic kidney disease; Ds, Disease; eGFR, estimated glomerular filtration rate by MDRD equation; KT, kidney transplantation; SE, standard error. ^a^ Generalized estimated equation analysis was performed. ^b^ Higher education was defined as receiving a diploma from college or higher. ^c^ Higher income was defined as monthly income above $ 4,500. *P* value by generalized estimated equation analysis.
